# Estimation and prediction of ellipsoidal molecular shapes in organic crystals based on ellipsoid packing

**DOI:** 10.1371/journal.pone.0239933

**Published:** 2020-09-30

**Authors:** Daiki Ito, Raku Shirasawa, Yoichiro Iino, Shigetaka Tomiya, Gouhei Tanaka

**Affiliations:** 1 Department of Electrical Engineering and Information Systems, Graduate School of Engineering, The University of Tokyo, Tokyo, Japan; 2 Materials Analysis Center, Fundamental Technology Research and Development Division 2, R&D Center, Sony Corporation, Atsugi, Japan; University of Salento, ITALY

## Abstract

Crystal structure prediction has been one of the fundamental and challenging problems in materials science. It is computationally exhaustive to identify molecular conformations and arrangements in organic molecular crystals due to complexity in intra- and inter-molecular interactions. From a geometrical viewpoint, specific types of organic crystal structures can be characterized by ellipsoid packing. In particular, we focus on aromatic systems which are important for organic semiconductor materials. In this study, we aim to estimate the ellipsoidal molecular shapes of such crystals and predict them from single molecular descriptors. First, we identify the molecular crystals with molecular centroid arrangements that correspond to affine transformations of four basic cubic lattices, through topological analysis of the dataset of crystalline polycyclic aromatic molecules. The novelty of our method is that the topological data analysis is applied to arrangements of molecular centroids intead of those of atoms. For each of the identified crystals, we estimate the intracrystalline molecular shape based on the ellipsoid packing assumption. Then, we show that the ellipsoidal shape can be predicted from single molecular descriptors using a machine learning method. The results suggest that topological characterization of molecular arrangements is useful for structure prediction of organic semiconductor materials.

## Introduction

Finding novel materials with desired properties often requires exhaustive search. In computational materials science, *ab initio* calculations based on density functional theory (DFT) have played a central role in analyzing physical properties of materials and testing the validity of experimental results. Although *ab initio* calculations are powerful, versatile, and efficient, they are still computationally expensive for several important classes of problems [1]. An alternative approach is materials informatics which exploits data science and informatics for reducing computational cost in material research [2, 3]. In particular, machine learning techniques have been increasingly leveraged to identify the hidden rules governing the structure-property-function relationship in materials from data. These methods have been successful in predicting material properties from atomistic and molecular information [4–12].

One of the challenging problems in materials science is crystal structure prediction (CSP). The goal of CSP is to accurately predict plausible crystal structures from atomistic and/or molecular information. The properties of molecular crystalline materials, such as energies and electronic characteristics, are highly sensitive to the arrangement of molecules due to complex intra- and inter-molecular interactions [13]. Therefore, CSP for molecular crystals is a significant step for materials property prediction [14]. Developing new effective computational methodologies for CSP is imperative for crystal engineering [15, 16], which aims to design crystalline materials with target structures leading to desired physical properties. However, even state-of-the-art computational methods for CSP require high computational cost for identifying plausible molecular arrangements which correspond to minimum energy structure [17].

From a geometrical viewpoint, some organic crystal structures are characterized by molecular packing [18, 19]. The structures of organic compounds with low-symmetry molecules have been analyzed under the close packing principle which implies that the minimum energies of compounds correspond to the packing structures of 3D molecular bodies with the least occupied volume [20–22]. The close packing principle and its modifications work well for many classes of organic crystals [23]. Whereas inorganic crystal structures composed of highly symmetric atomic bodies are often explained by close packing of spheres, organic crystal structures consisting of low-symmetry molecular bodies are linked to dense packing of ellipsoids [24, 25].

Many organic semiconductor materials are involved in aromatic systems. Motivated by this fact, we focus on the dataset of polycyclic aromatic molecules which are used to assess the effectiveness of our method. We first employ topological data analysis to identify the organic molecular crystals where the arrangement of molecular centroids coincides with affine transformations of basic cubic lattices. Then, we estimate the shapes of ellipsoids packed in the identified organic crystals under the ellipsoid packing assumption. Moreover, we show that the ellipsoidal shapes can be predicted from single molecular descriptors using a machine learning method for the dataset of polycyclic aromatic molecules. The ellipsoid radii can correspond to the approximate intracrystalline molecular shape, and therefore, our method is useful for predicting partial information of crystal structures.

## Methods

### Overview

**Fig 1:**
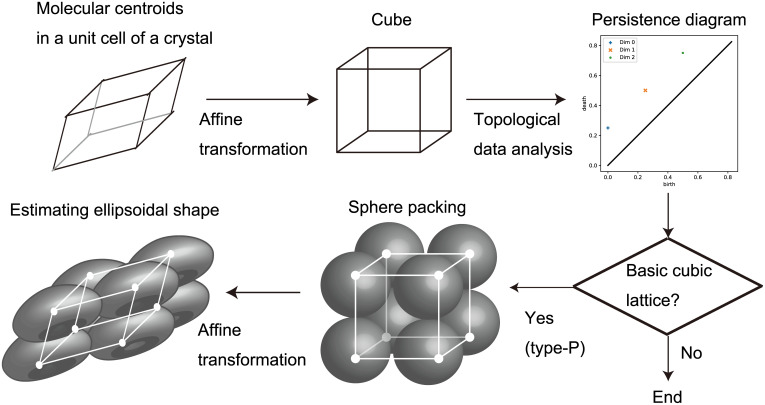
Methodology overview. An overview of the method to estimate the shapes of ellipsoids packed in molecular crystals is illustrated. This is an example for an organic molecular crystal where the molecular arrangement corresponds to the primitive (type-P) lattice (see Figs 2(a) and 4(a) for details).

An overview of our method for estimating ellipsoidal molecular shapes is shown in Fig 1. Our method relies on the concept of ellipsoid packing for organic crystal structures. We generate a persistence diagram from an arrangement of molecular centroids. If the generated persistence diagram coincides with a theoretically derived diagram for a basic cubic lattice, then we can estimate the ellipsoidal shapes from the identified lattice type and the specified affine transformation. The estimated ellipsoidal shape can be an approximate representation of an intracrystalline molecular shape, which is predicted from single molecular descriptors using a machine learning technique.

### Ellipsoid packing

Crystal structures have been interpreted in terms of packing of atoms and molecules in crystallography. Some inorganic crystal structures have been explained as close packing of spheres which approximate atomic bodies with high symmetry. The two models that achieve dense packing of identical spheres are known to be cubic closest packing (ccp) and hexagonal closest packing (hcp) [21, 22]. The structure of ccp is also known as a face-centered cubic (fcc) lattice in the cubic crystal system. For both ccp and hcp, the packing fraction is given by $\rho =\pi /\sqrt{18}∼0.74048$. Sphere packing itself has a long history in mathematics [26]. As for organic crystals, the constituent units are molecules with less symmetry and their bodies are suitably approximated by ellipsoids rather than spheres. Ellipsoid packing is an extended problem of sphere packing [27]. It was reported that densest packing structures of identical ellipsoids are limited to affine transformations of closest packing structures of identical spheres [24].

**Fig 2:**
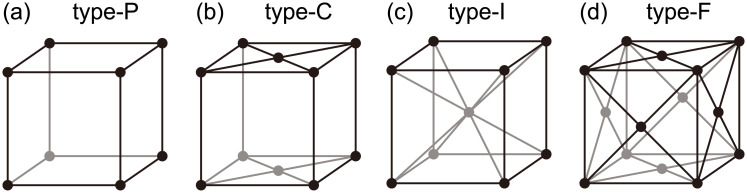
Basic cubic lattices. (a) Primitive (type-P) lattice. (b) Base-centered (type-C) lattice. (c) Body-centered (type-I) lattice. (d) Face-centered (type-F) lattice.

In this study, we deal with organic crystal structures from the viewpoint of ellipsoid packing following the above report, although an unusual case of densest crystal ellipsoid packing was later found in the glassy phases [28] and also in crystal packing. Molecular arrangements are determined by arrangements of centroids of molecules. We limit our focus to the crystals with molecular arrangements that are obtained by affine transformations of the cubic lattices illustrated in Fig 2. Fig 2(a)–2(d) correspond to the basic lattices, called Primitive (type-P), Base-centered (type-C), Body-centered (type-I), and Face-centered (type-F), respectively, in crystal systems. Affine transformations of these cubic lattices cover all the crystal families except for the hexagonal family. The hexagonal family is not considered in this study because the targeted crystal structure dataset does not contain crystal structures corresponding to its affine transformations. We first convert a unit cell of a molecular crystal to a cube by an affine transformation and then analyze it using persistent homology.

### Topological data analysis

Topological data analysis is an emerging mathematical technology to analyze topological properties of structural data using applied algebraic topology and computational geometry [29–31]. It enables to characterize qualitative features of a set of discrete points in space. Persistent homology is a powerful framework for topological data analysis [32, 33], which can reveal topological properties of a point cloud (i.e. a set of data points) at different spatial resolutions and generate a persistence diagram (i.e. a visualization of persistent homology as a 2D histogram).

**Fig 3:**
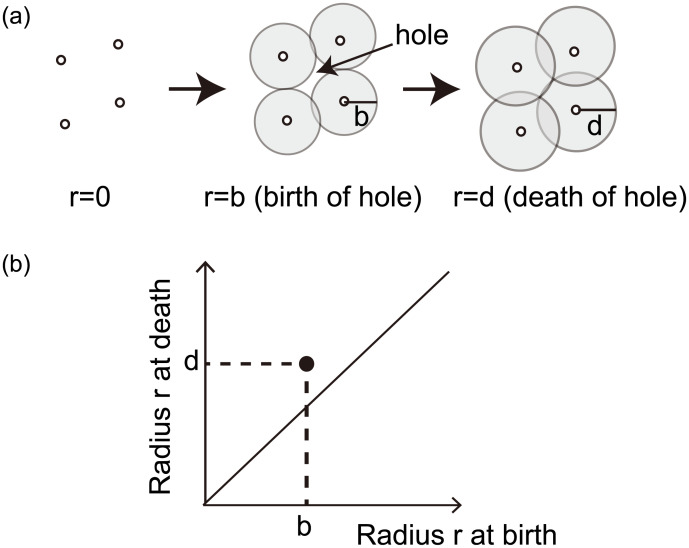
An example of persistent homology. (a) Filtration for a point cloud on a 2D space. (b) Persistence diagram.

Fig 3(a) illustrates an example of filtration for a point cloud on a 2D space. We consider disks with radius *r* > 0, centered at the four data points. As *r* is increased from 0, the initially separated disks start to overlap with each other at a certain value of *r*. The change in the *r* value means a change in the resolution. If the union of disks makes a hole at *r* = *b* and the hole vanishes at *r* = *d* as in Fig 3(a), then the birth and death of the hole are recorded as a point at (*b*, *d*) in the persistence diagram as shown in Fig 3(b). Such points are plotted with respect to each hole that appears when continuously increasing the disk size. Plotted points in a persistence diagram exist above the diagonal line, because a death of a hole occurs after its birth. Topologically similar point clouds produce similar persistence diagrams.

The persistence diagram for a point cloud $P=\{{\text{p}}_{i}\in R^{3}\;|\;i=1,\ldots ,I\}$ consisting of *I* discrete points in a 3D space is defined as follows:

$$\begin{array}{ccc} D_{q}\left(P\right) & = & \{\left(b_{j},d_{j}\right)\in R^{2}\;|\;j=1,\ldots ,J\}, \end{array}$$ (1)

where *J* is the number of holes and *q* is the dimensionality of holes, e.g. *q* = 0 for connected components, *q* = 1 for rings, and *q* = 2 for cavities. We use the multi-set {*D*_*q*_(*P*) | *q* = 0, 1, 2} to characterize a topological feature of the point cloud *P*.

We analytically derived the persistence diagrams for the four cubic lattices shown in Fig 2, where the length of each side is given by *α*. Denoting the point clouds for type-P, type-C, type-I, and type-F lattices by *P*_P_, *P*_C_, *P*_I_, and *P*_F_, respectively, we obtain the following results:

- For type-P,
  $$\begin{array}{ccc} D_{0}\left(P_{P}\right) & = & \{(0,\alpha /2),(0,\infty )\},\\ D_{1}\left(P_{P}\right) & = & \{\left(\alpha /2,\alpha /\sqrt{2}\right)\},\\ D_{2}\left(P_{P}\right) & = & \{\left(\alpha /\sqrt{2},\sqrt{3}\alpha /2\right)\}. \end{array}$$ (2)
- For type-C,
  $$\begin{array}{ccc} D_{0}\left(P_{C}\right) & = & \{\left(0,\alpha /2\sqrt{2}\right),\left(0,\alpha /2\right),\left(0,\infty \right)\},\\ D_{1}\left(P_{C}\right) & = & \{\left(\alpha /2\sqrt{2},\alpha /2\right),\left(\alpha /2,\sqrt{3}\alpha /2\sqrt{2}\right)\},\\ D_{2}\left(P_{C}\right) & = & \{\left(\sqrt{3}\alpha /2\sqrt{2},\alpha /\sqrt{2}\right)\}. \end{array}$$ (3)
- For type-I,
  $$\begin{array}{ccc} D_{0}\left(P_{I}\right) & = & \{\left(0,\sqrt{3}\alpha /4\right),\left(0,\infty \right)\},\\ D_{1}\left(P_{I}\right) & = & \{\left(\sqrt{3}\alpha /4,3\alpha /4\sqrt{2}\right),\left(\alpha /2,3\alpha /4\sqrt{2}\right)\},\\ D_{2}\left(P_{I}\right) & = & \{\left(3\alpha /4\sqrt{2},\sqrt{5}\alpha /4\right)\}. \end{array}$$ (4)
- For type-F,
  $$\begin{array}{ccc} D_{0}\left(P_{F}\right) & = & \{\left(0,\alpha /2\sqrt{2}\right),\left(0,\infty \right)\},\\ D_{1}\left(P_{F}\right) & = & \{\left(\alpha /2\sqrt{2},\alpha /\sqrt{6}\right)\},\\ D_{2}\left(P_{F}\right) & = & \{\left(\alpha /\sqrt{6},\sqrt{3}\alpha /4\right),\left(\alpha /\sqrt{6},\alpha /2\right)\}. \end{array}$$ (5)

**Fig 4:**
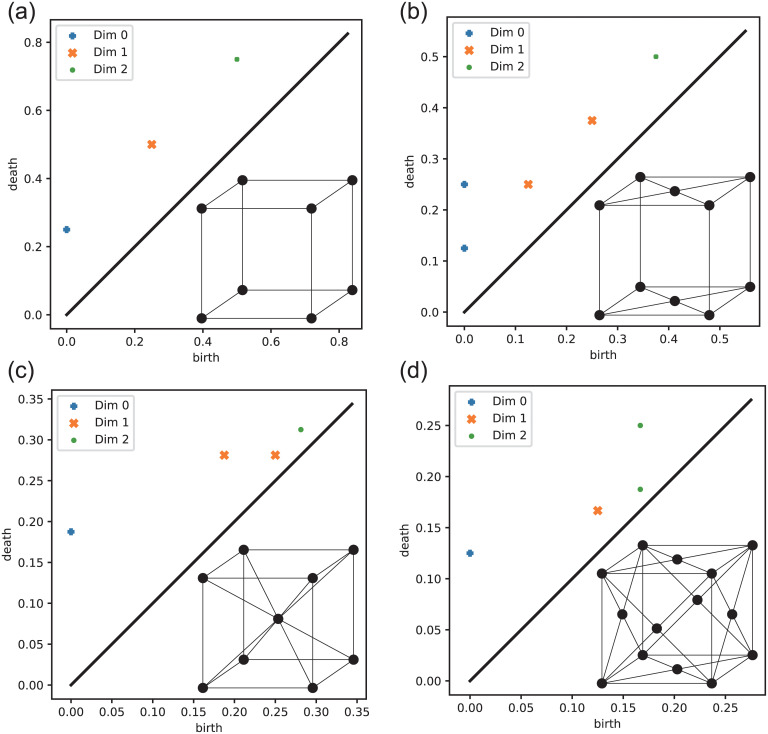
Persistent diagrams for the cubic lattices in Fig 2. In each panel, the blue pluses, orange crosses, and green dots indicate points for *q* = 0, 1, 2. In the right-bottom part of each panel, the corresponding lattice structures are shown. (a) type-P (Eq (2)). (b) type-C (Eq (3)). (c) type-I (Eq (4)). (d) type-F (Eq (5)).

The corresponding persistence diagrams are shown in Fig 4(a)–4(d).

The similarity between two persistence diagrams, *X* and *Y*, can be measured with the following bottleneck distance [29]:

$$\begin{array}{ccc} d_{B}\left(X,Y\right) & = & \underset{\eta :X\to Y}{\text{inf}}\underset{x\in X}{\text{sup}}||x-{\eta \left(x\right)||}_{\infty }, \end{array}$$ (6)

where *η* is a bijection between *X* and *Y*, and the *L*_∞_-distance between points *u* = (*u*_1_, *u*_2_) and *v* = (*v*_1_, *v*_2_) is defined as follows:

$$\begin{array}{c} ||u-{v||}_{\infty }=\text{max}\{|u_{1}-v_{1}\left|,\right|u_{2}-v_{2}\left|\right\}. \end{array}$$ (7)

In short, the bottleneck distance is the cost of the optimal matching between points of the two diagrams.

Using this similarity measure, we identify the crystals where the molecular arrangements correspond to affine transformations of the cubic lattices in Fig 2. First, a unit cell of a crystal, represented as a parallelepiped, is converted to a cube with each side *α* = 0.1 nm by an affine transformation. This normalization transformation is necessary because a persistence diagram is robust against a rotation of the targeted point cloud and noise but largely affected by a difference in the scales. Then, we compute persistence diagrams *D*_*q*_(*P*_M_) (*q* = 0, 1, 2) for a point cloud *P*_M_ in the cube, corresponding to a set of molecular centroids. If the multi-set *D*_*q*_(*P*_M_) is sufficiently close to one of those in Eqs (2)–(5), then the arrangement of molecular centroids is categorized into the same affine group. The persistence diagram for *P*_M_ is considered to be equivalent to that of *P*_M_ (Z = P, C, I, or F), if the following inequality is satisfied:

$$\begin{array}{c} \min_{q=0,1,2}d_{B}\left(D_{q}\left(P_{M}\right),D_{q}\left(P_{Z}\right)\right)<\epsilon , \end{array}$$ (8)

where *ϵ* represents the acceptable error. In later numerical experiments, we set *ϵ* at 0.001 nm. For numerically generating persistence diagrams for molecular centroids, we used Dionysus 2 which is a library for computing persistent homology [34].

### Estimation method of ellipsoidal shapes

Once we identify a crystal that has a structure corresponding to an affine transformation of a cubic lattice, we can estimate the shape of identical ellipsoids around the molecular centroids packed in the identified cubic lattice. In general, an ellipsoid is expressed as follows:

$$\begin{array}{ccc} {\text{x}}^{⊤}R\text{x} & = & 1, \end{array}$$ (9)

where **x** = (*x*_1_, *x*_2_, *x*_3_)^⊤^ is a 3D coordinate. The diagonal matrix *R* is represented as follows:

$$\begin{array}{c} R=[\begin{array}{ccc} r_{1}^{-2} & 0 & 0\\ 0 & r_{2}^{-2} & 0\\ 0 & 0 & r_{3}^{-2} \end{array}], \end{array}$$ (10)

where *r*_*i*_ denotes the radius in the direction of *x*_*i*_ for *i* = 1, 2, 3.

For ellipsoids (or spheres) packed in the four cubic lattices, the diagonal matrices *R*_Z_ for Z = P, C, I, or F are calculated as follows:

$$\begin{array}{ccc} R_{P} & = & [\begin{array}{ccc} {\left(\alpha /2\right)}^{-2} & 0 & 0\\ 0 & {\left(\alpha /2\right)}^{-2} & 0\\ 0 & 0 & {\left(\alpha /2\right)}^{-2} \end{array}], \end{array}$$ (11)

$$\begin{array}{ccc} R_{C} & = & [\begin{array}{ccc} {\left(\sqrt{2}\alpha /4\right)}^{-2} & 0 & 0\\ 0 & {\left(\sqrt{2}\alpha /4\right)}^{-2} & 0\\ 0 & 0 & {\left(\alpha /2\right)}^{-2} \end{array}], \end{array}$$ (12)

$$\begin{array}{ccc} R_{I} & = & [\begin{array}{ccc} {\left(\sqrt{3}\alpha /4\right)}^{-2} & 0 & 0\\ 0 & {\left(\sqrt{3}\alpha /4\right)}^{-2} & 0\\ 0 & 0 & {\left(\sqrt{3}\alpha /4\right)}^{-2} \end{array}], \end{array}$$ (13)

$$\begin{array}{ccc} R_{F} & = & [\begin{array}{ccc} {\left(\sqrt{2}\alpha /4\right)}^{-2} & 0 & 0\\ 0 & {\left(\sqrt{2}\alpha /4\right)}^{-2} & 0\\ 0 & 0 & {\left(\sqrt{2}\alpha /4\right)}^{-2} \end{array}], \end{array}$$ (14)

where *α* is the length of each side of the unit cell.

Now we assume that an ellipsoid approximating a molecular shape in a crystal is represented as follows:

$$\begin{array}{ccc} {\text{v}}^{⊤}Q\text{v} & = & 1 \end{array}$$ (15)

where **v** is a 3D coordinate and *Q* is an unknown diagonal matrix determining the shape of the ellipsoid. We denote by *W* the affine transformation used to normalize the unit cell of a crystal when generating the persistence diagram. Then the coordinate transformation is represented as **x** = *W* **v**. The ellipsoid in Eq (15) is transformed by *W* into the ellipsoid **x**^⊤^ *R*_Z_ **x** = 1 with a known diagonal matrix *R*_Z_ (Z = P, C, I, or F). By substituting **x** = *W* **v** into **x**^⊤^ *R*_Z_ **x** = 1, we obtain

$$\begin{array}{c} v^{⊤}\left(W^{⊤}R_{Z}W\right)v=1, \end{array}$$ (16)

where *W*^⊤^ *R*_Z_ *W* is a symmetric matrix. The radii *r*_*i*_ (*i* = 1, 2, 3) of the ellipsoid approximating the intracrystalline molecular shape are computed as $r_{i}=1/\sqrt{\lambda_{i}}$, where λ_*i*_ (*i* = 1, 2, 3) represent the eigenvalues of *W*^⊤^ *R*_Z_ *W*.

### Prediction method of ellipsoidal shapes

The above-mentioned method gives approximate ellipsoidal molecular shapes represented as ellipsoid radii. We test whether these ellipsoidal shapes can be predicted from single molecular descriptors using a machine learning method. Molecular descriptors represent a set of features of single molecules, which have many possible representations. Molecular fingerprint is one of the widely used descriptors to determine the similarity of chemical structures [35, 36]. A molecular fingerprint is represented as a binary feature vector where each component expresses whether an attribute is present or absent in the molecule.

We employ Extended Connectivity Fingerprint (ECFP) [37] which is one of the data-driven circular fingerprints unlike those based on predefined substructural keys. A molecule is represented as a graph where the vertices are atoms and the edges are bonds. Subgraphs included in the neighborhood of each vertex up to a fixed diameter are examined and quantified with the atomic features using the Morgan algorithm [38]. Then these substructural features are mapped into integer codes using a hashing procedure to keep the length of the feature vector fixed. The ECFP is obtained as a binary feature vector from the resulting identifiers. For converting molecular information into ECFPs, we used Chainer Chemistry which is an open-source library for deep learning in biology and chemistry [39].

The ellipsoidal shape prediction is performed with a feedforward neural network with one hidden layer in a supervised learning framework. The hidden layer has 64 units. Each node has the hyperbolic tangent (tanh) activation function. The number of training data is denoted by *N*. The *n*th teacher data is given by a pair of input and output, where the input is an ECFP for the molecule and the output is the estimated ellipsoid radii $r_{1}^{\left(n\right)}$, $r_{2}^{\left(n\right)}$, and $r_{3}^{\left(n\right)}$ for the corresponding molecular crystal. We train a neural network model so as to minimize the mean squared error between the network output ${\hat{r}}_{i}^{\left(n\right)}$ and the teacher output $r_{i}^{\left(n\right)}$, described as follows:

$$\begin{array}{ccc} E & = & \frac{1}{2N}\sum_{n=1}^{N}\sum_{i=1}^{3}({\hat{r}}_{i}^{\left(n\right)}-r_{i}^{\left(n\right)}{)}^{2}. \end{array}$$ (17)

Using a test dataset, we evaluate the prediction accuracy in predicting the ellipsoidal shape.

## Results

### Dataset

Molecular crystals are versatile materials which can be found in pharmaceuticals, organic semiconductors, solid-state reactions, and plastic materials [40]. For instance, polycyclic aromatic hydrocarbons (PAHs) and their derivatives have been widely explored for organic semiconductors [41]. Such organic molecular crystals are important targets of CSP because their molecular arrangements are deeply involved in electron mobility in the crystals [42]. We selected polyaromatic crystals that consist of only one type of molecule from the Cambridge Structural Database (CSD) provided by Cambridge Crystallographic Data Centre (CCDC) [43]. We made the following three crystal datasets:

- Polycyclic aromatic hydrocarbons (PAHs): 75 crystals of polycyclic aromatic hydrocarbons that contain only hydrogens and carbons.
- Polycyclic aromatics with hetero atoms (PAHAs): 404 crystals of polycyclic aromatic hydrocarbons that can contain hetero elements (N, S, etc.) in their skeletons but have no substituents other than halogens.
- Polycyclic aromatics (PAs): 8787 crystals of polycyclic aromatic hydrocarbons that can contain hetero elements in their skeletons and arbitrary substituents.

**Fig 5:**
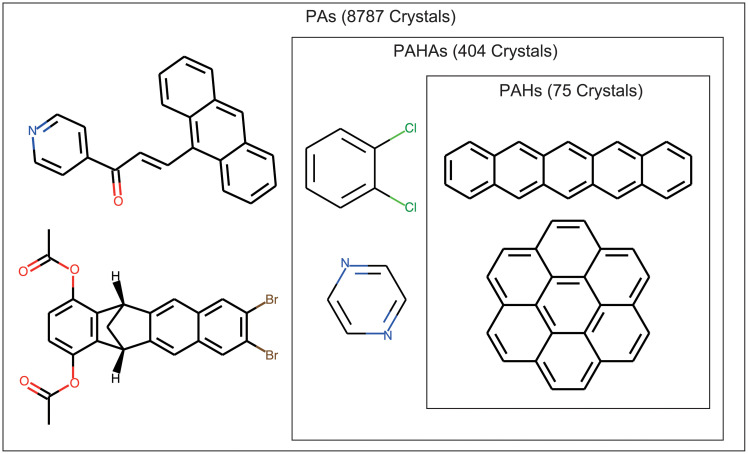
Dataset of organic molecular crystals. The three datasets correspond to polycyclic aromatic hydrocarbons (PAHs), polycyclic aromatics with hetero atoms (PAHAs), and polycyclic aromatics (PAs).

PAHs are included in PAHAs which are included in PAs, as shown in Fig 5.

### Identification of crystals with specific structures

The topological data analysis was applied to molecular centroids of each crystal structure in the datasets. It was determined whether each crystal structure corresponds to an affine transformation of one of the four cubic lattices shown in Fig 2. Table 1 shows the numbers of organic crystals that were classified into the four types (i.e. type-P, type-C, type-I, and type-F) for each dataset. The others were categorized into the class of “Others.” The fraction of crystals corresponding to the four types to the total number of crystals in the PAHs is about 30%, that in the PAHAs is about 21%, and that in the PAs is about 7%. The high fraction of simple crystal structures in the PAHs is related to the fact that some crystals in the PAHs tend to have layered molecular arrangements and their structures are classified into typical packing motifs [44, 45]. It also implies that the crystal structure is complex when hetero atoms are contained in molecules as in the PAHAs and PAs.

**Table 1:** Identification of crystals corresponding to affine transformed cubic lattices.

	PAHs	PAHAs	PAs
**type-P**	0	0	160
**type-C**	9	34	205
**type-I**	8	34	153
**type-F**	6	15	104
**Others**	52	321	8165
**Total**	75	404	8787

**Fig 6:**
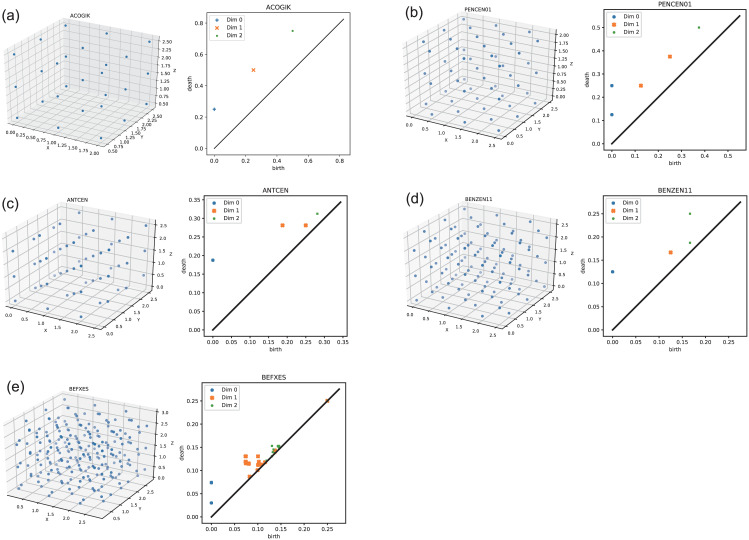
Examples of persistence diagrams for organic crystals. Molecular centroids (left) and corresponding persistence diagrams (right). In each persistence diagram, the blue pluses, orange crosses, and green circles indicate the plots of *D*_*q*_ with *q* = 0, *q* = 1, and *q* = 2, respectively. (a) 9,10-bis(2-(4-(n-Decyloxy)phenyl)vinyl)anthracene (PAs) with type-P structure. (b) Pentacene (PAHs) with type-C structure. (c) Anthracene (PAHs) with type-I structure. (d) Benzene (PAHs) with type-F structure. (e) Acenaphthobenzopicene (PAHs) which is categorized into “Others”.

Fig 6 illustrates the examples of molecular centroid arrangements and the corresponding persistence diagrams for the crystals categorized in the five types (see Table 1). Fig 6(a)–6(d) show 9,10-bis(2-(4-(n-Decyloxy)phenyl)vinyl)anthracene with type-P structure, Pentacene with type-C structure, Anthracene with type-I structure, and Benzene with type-F structure, respectively. In other words, these persistence diagrams are found in Fig 4. Fig 6(e) shows Acenaphthobenzopicene which has a lower symmetric structure than the four other crystal structure types as seen from the dispersion of points in the persistence diagram.

### Ellipsoidal shape estimation

**Fig 7:**
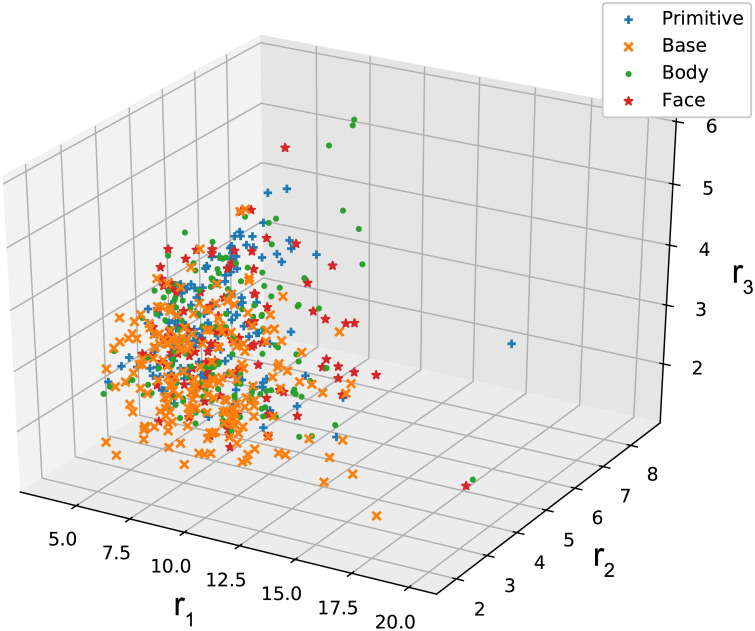
Estimated radii of ellipsoids. Each plot at (*r*_1_, *r*_2_, and *r*_3_) indicates the radii of ellipsoids packed in a crystal that was identified in Table 1. The blue pluses, orange crosses, green circles, and red stars correspond to type-P, type-C, type-I, and type-R, respectively.

**Fig 8:**
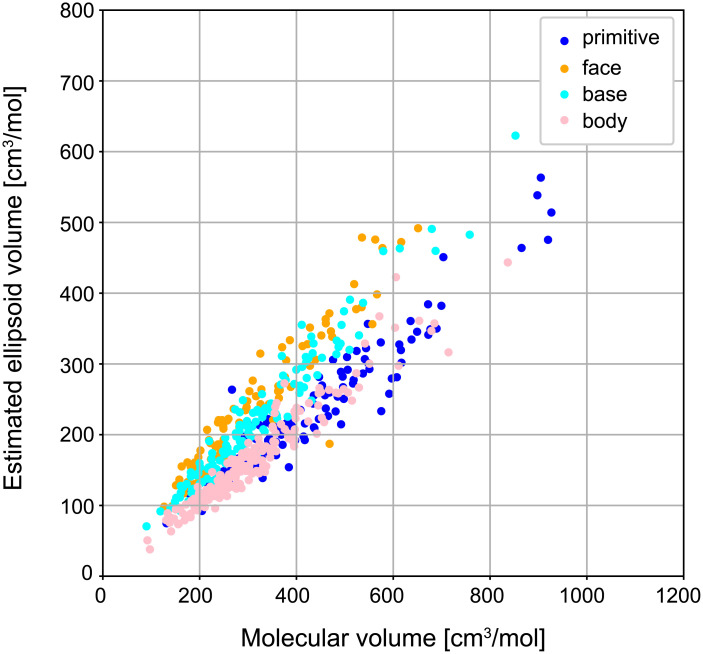
Correlation between the estimated ellipsoid volume and the molecular volume. The ellipsoid volume [cm^3^/mol] (the vertical axis) was calculated from the estimated radii. The molecular volume [cm^3^/mol] (the horizontal axis) was calculated using Gaussian 16 which is a general purpose computational chemistry software [46]. The Pearson’s correlation coefficient for all the points is 0.897.

We assume that identical ellipsoids around the molecular centroids are packed in the crystals that were identified in Table 1. The ellipsoidal shape was estimated from Eq (16). Fig 7 shows the distribution of the estimated ellipsoidal radii for the PAs that correspond to the affine transformations of the basic cubic lattices. The three axes indicate *r*_1_, *r*_2_, and *r*_3_, satisfying *r*_1_ ≥ *r*_2_ ≥ *r*_3_. The different marks correspond to different types of cubic lattices in Fig 2. To validate the above assumption, we investigated a correlation between the ellipsoidal volume *V* = (4/3)*πr*_1_ *r*_2_ *r*_3_ calculated from the estimated radii and the molecular volume calculated based on the electron density through Monte-Carlo integration in Gaussian 16 [46]. The results are shown in Fig 8. The molecular structures and the electron densities were calculated by DFT calculation at the theoretical level of B3LYP with 6-31G basis set. The result shows a high correlation (the Pearson’s correlation coefficient: 0.897), indicating that the ellipsoidal volume well approximates the molecular volume. Similarly, we obtained the estimated ellipsoid radii for the two other datasets, PAHAs and PAHs (not shown).

**Fig 9:**
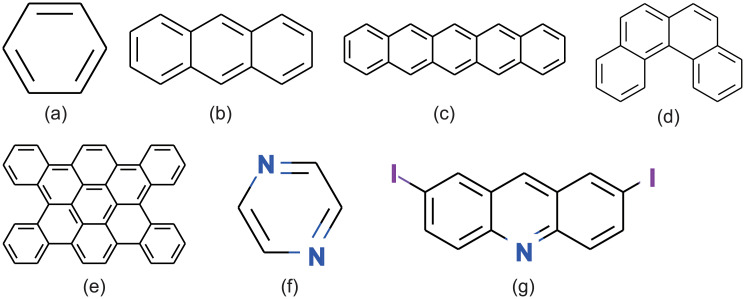
Structures of single isolated molecules in Table 2. (a) Benzene. (b) Anthracene. (c) Pentacene. (d) Benzophenanthrene. (e) Tetrabenzocoronene. (f) Pyrazine. (g) 2,7-bisacridine.

The estimated ellipsoid radii for some crystals are listed in Table 2. The structures of the molecules listed in Table 2(a)–2(g) are shown in Fig 9(a)–9(g). It shows that the ellipsoidal shapes are affected by the single molecular structures. For example, Benzene (Fig 9(a)), Anthracene (Fig 9(b)), and Pentacene (Fig 9(c)) have one, three, and five aromatic rings in a series, respectively. As the number of aromatic rings increases, only the estimated radius *r*_1_ tends to be elongated. Benzophenanthrene (Fig 9(d)) and Tetrabenzocoronene (Fig 9(e)) have planar disk-like structures, and thus, the estimated values of *r*_1_ and *r*_2_ are relatively large compared with that of *r*_3_. While the single molecular structure is a major factor affecting the ellipsoid radii, its chemical structure also influences them. The shapes of Pyrazine (Fig 9(f)) and 2,7-bisacridine (Fig 9(g)) look similar to the shapes of Benzen and Anthracene, respectively, but *r*_1_ is longer due to the influence of terminal hetero atoms.

**Table 2:** Examples of estimated ellipsoid radii.

Molecules	*r*_1_	*r*_2_	*r*_3_
**(a) Benzene**	0.3253 nm	0.2576 nm	0.2365 nm
**(b) Anthracene**	0.5469 nm	0.2699 nm	0.2699 nm
**(c) Pentacene**	0.7142 nm	0.2707 nm	0.2190 nm
**(d) Benzophenanthrene**	0.5183 nm	0.5005 nm	0.2045 nm
**(e) Tetrabenzocoronene**	0.8702 nm	0.6280 nm	0.1630 nm
**(f) Pyrazine**	0.4041 nm	0.2532 nm	0.1634 nm
**(g) 2,7-bisacridine**	0.9566 nm	0.2483 nm	0.2144 nm

### Ellipsoidal shape prediction

**Fig 10:**
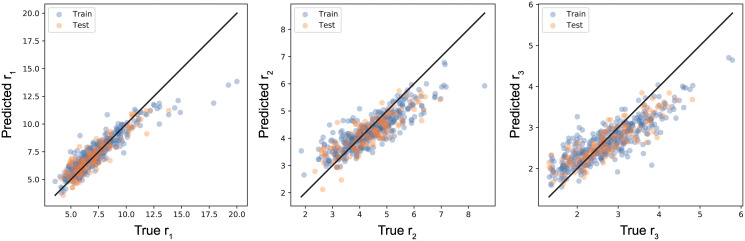
Prediction of the ellipsoid radii for PAs from ECFPs. The horizontal axis represents the teacher data and the vertical axis represents the predicted value. (a) *r*_1_. (b) *r*_2_. (c) *r*_3_.

We performed a machine learning prediction of the ellipsoidal radii for PAs from single molecular information. This is regarded as a simplified task of CSP, because the information of arrangements of molecular centroids and molecular shapes are useful for identifying crystal structures. The task is to predict the radii of ellipsoids (*r*_1_, *r*_2_, and *r*_3_) from molecular fingerprints given by ECFPs. We trained a neural network model by using the Adam (Adaptive moment estimation) optimizer with learning rate 0.001 and mini-batch size 32 [47]. We used 10% of the training data for early stopping to avoid overfitting and evaluated our model via four-fold cross validation. The results of the ellipsoid radii prediction are shown in Fig 10. In each panel, The horizontal axis represents the true radius and the vertical axis represents the predicted one. The plots for the *r*_1_ prediction are close to the diagonal line as shown in Fig 10(a), implying a successful prediction. The mean training error is around 0.04 nm and the testing error is around 0.094 nm. On the other hand, we can find that the predicted values for *r*_2_ and *r*_3_ tend to be smaller than the actual values when their values are large as shown in Fig 10(b) and 10(c). The prediction results for PAHs and PAHAs are shown in S1 Fig. The results suggest that our method is useful for predicting the length of the main axis of the skeleton in organic semiconductor materials rather than lengths of the shorter ellipsoid half-axes. As seen in Table 2, the ellipsoid radii are influenced not only by the single molecular shape but also by the chemical constitution. Therefore, the molecular fingerprints including such information worked well in the ellipsoidal shape prediction. We can choose other machine learning models for the radii prediction and an improvement in the prediction performance is an issue to be considered.

## Conclusion and discussion

We have studied the problem of ellipsoid estimation and prediction in terms of molecular packing, mainly targeted for polycyclic aromatics found in organic semiconductor materials. We have identified the organic molecular crystals whose structures are characterized by affine transformations of the four basic cubic lattices, through the topological analysis of the dataset of molecular centroids in crystals of aromatic molecules. Then, we have computed the radii of ellipsoids around the molecular centroids of those crystals from the identified lattice type and the specified affine transformation under the dense packing assumption. The ellipsoid shape represents an approximate shape of the intracrystalline molecules, which provides partial information of molecular structures in crystals. In additional experiments, we have shown that the ellipsoid radii can be predicted from the single molecular descriptors. Our results suggest that a combination of topological data analysis and machine learning can partly contribute to CSP.

It has been known that some molecular crystal structures are explained by ellipsoid packing corresponding to minimum energy structure. However, it was not straightforward to automatically identify such crystals from molecular centroid data because of the difficulty in checking coincidence of point groups in a 3D space. Thus, we have used the persistent homology to transform the point arrangement in the 3D space into the 2D persistence diagram. This method is useful particularly when checking the topological similarity between molecular arrangements. Our experiments have shown that some molecular arrangements (centroids) can be classified into basic lattices via affine transformations. This is analogous to the fact that spatial arrangements of atoms in crystals are characterized by Bravais lattices. A further study on topological classification of molecular arrangements would be effective for understanding molecular structures in crystals.

The focus of our study has been limited to the crystals with specific structures obtained by affine transformations of the basic cubic lattices, which cover triclinic, monoclinic, orthorhombic, tetragonal, and cubic crystal families. The remaining family is the hexagonal crystal family. We have excluded this family in this study due to the difficulty in analytical derivation of the corresponding persistence diagram and the ellipsoid radii under the dense packing condition. A future work is to extend our method such that the crystals having structures corresponding to affine transformations of hexagonal lattices are also handled. Another issue is that the affine transformation restricts the molecular orientations. To distinguish diverse orientations such as a herringbone motif, it would be necessary to apply other transformations between molecular centroids and basic lattices.

The proposed method has several limitations. First, it does not give information about the arrangements of atoms in each crystalline molecule. For a full CSP, a conformational search of the arrangements of atoms after applying the proposed method would be necessary. Second, the assumptions in our method are applicable only to a part of crystal structures. For instance, the approximation of molecules with ellipsoids would be valid for rigid molecules but not for highly flexible molecules. A possible approach for examining how much molecular flexibility is permitted is to investigate the effect of the number of dihedral angles, representing the flexibility level, on the prediction accuracy. The dense packing assumption would also not be applicable to structures with substituents such as -CHO hydrogen bond, because they can be low-density structures. For such structures, *ab initio* calculations with force fields and DFT would be appropriate [48]. Third, the fraction of the number of structures identified based on our assumption is not large as shown in Table 1 for the dataset used in this study. This might be caused by the smallness of the acceptable error *ϵ* in Eq (8), which is a severe condition for determining identical persistence diagrams. There is a possibility that the fraction is increased by increasing the value of the acceptable error *ϵ* in Eq (8), but there would still be remaining structures that are not identified by our assumption. To extend the applicability of our method, we need to consider other lattice types in addition to those in Fig 2.

## Supporting information

S1 Fig Prediction of the ellipsoid radii for PAHs and PAHAs from ECFPs. The horizontal axis represents the teacher data and the vertical axis represents the predicted value. (a)-(c) the prediction results on *r*_1_, *r*_2_, and *r*_3_ for PAHs. (d)-(f) the prediction results on *r*_1_, *r*_2_, and *r*_3_ for PAHAs.
(EPS)
